# Epidemiology of gestational diabetes mellitus according to IADPSG/WHO 2013 criteria among obese pregnant women in Europe

**DOI:** 10.1007/s00125-017-4353-9

**Published:** 2017-07-12

**Authors:** Aoife M. Egan, Akke Vellinga, Jürgen Harreiter, David Simmons, Gernot Desoye, Rosa Corcoy, Juan M. Adelantado, Roland Devlieger, Andre Van Assche, Sander Galjaard, Peter Damm, Elisabeth R. Mathiesen, Dorte M. Jensen, Liselotte Andersen, Annuziata Lapolla, Maria G. Dalfrà, Alessandra Bertolotto, Urszula Mantaj, Ewa Wender-Ozegowska, Agnieszka Zawiejska, David Hill, Judith G. M. Jelsma, Frank J. Snoek, Christof Worda, Dagmar Bancher-Todesca, Mireille N. M. van Poppel, Alexandra Kautzky-Willer, Fidelma P. Dunne, on behalf of the DALI Core Investigator group

**Affiliations:** 1 Galway Diabetes Research Centre, College of Medicine, Nursing and Health Sciences, National University of Ireland, University Road, Galway, H91 TK33 Ireland; 2 Gender Medicine Unit, Division of Endocrinology and Metabolism, Department of Medicine III, Medical University of Vienna, Vienna, Austria; 3 Institute of Metabolic Science, Addenbrookes Hospital, Cambridge, UK; 4 Macarthur Clinical School, Western Sydney University, Sydney, NSW Australia; 5 Department of Obstetrics and Gynecology, Medizinische Universitaet Graz, Graz, Austria; 6 Institut de Recerca de l’Hospital de la Santa Creu i Sant Pau, Barcelona, Spain; 7 CIBER Bioengineering, Biomaterials and Nanotechnology, Instituto de Salud Carlos III, Zaragoza, Spain; 8 KU Leuven Department of Development and Regeneration: Pregnancy, Fetus and Neonate, University Hospitals Leuven, Leuven, Belgium; 9 Gynaecology and Obstetrics, University Hospitals Leuven, Leuven, Belgium; 10 Department of Obstetrics and Gynaecology, Division of Obstetrics and Prenatal Medicine, Erasmus MC, University Medical Centre Rotterdam, Rotterdam, the Netherlands; 11 Center for Pregnant Women with Diabetes, Departments of Endocrinology and Obstetrics, Rigshospitalet, Copenhagen, Denmark; 12 Institute of Clinical Medicine, Faculty of Health and Medical Sciences, University of Copenhagen, Copenhagen, Denmark; 13 Department of Endocrinology, Odense University Hospital, Faculty of Health Science, University of Southern Denmark, Odense, Denmark; 14 Department of Gynaecology and Obstetrics, Odense University Hospital, Faculty of Health Science, University of Southern Denmark, Odense, Denmark; 15 Department of Clinical Research, Faculty of Health Science, University of Southern Denmark, Odense, Denmark; 16 Universita Degli Studi di Padova, Padua, Italy; 17 Azienda Ospedaliero-Universitaria Pisana, Pisa, Italy; 18 Medical Faculty I, Poznan University of Medical Sciences, Poznan, Poland; 19 Recherche en Santé Lawson SA, St Gallen, Switzerland; 20 Department of Public and Occupational Health, EMGO+ Institute for Health and Care Research, VU University Medical Centre, Van der Boechorststraat 7, 1081 BT Amsterdam, the Netherlands; 21 Department of Medical Psychology, EMGO+ Institute for Health and Care Research, VU University Medical Centre and Medical Psychology AMC, Amsterdam, the Netherlands; 22 Division of Obstetrics and Feto-Maternal Medicine, Department of Obstetrics and Gynecology, Medical University of Vienna, Vienna, Austria; 23 Institute of Sports Science, University of Graz, Graz, Austria

**Keywords:** Clinical diabetes, Clinical science and care, Epidemiology, Healthcare delivery, Pregnancy, Weight regulation and obesity

## Abstract

**Aims/hypothesis:**

Accurate prevalence estimates for gestational diabetes mellitus (GDM) among pregnant women in Europe are lacking owing to the use of a multitude of diagnostic criteria and screening strategies in both high-risk women and the general pregnant population. Our aims were to report important risk factors for GDM development and calculate the prevalence of GDM in a cohort of women with BMI ≥29 kg/m^2^ across 11 centres in Europe using the International Association of the Diabetes and Pregnancy Study Groups (IADPSG)/WHO 2013 diagnostic criteria.

**Methods:**

Pregnant women (*n* = 1023, 86.3% European ethnicity) with a BMI ≥29.0 kg/m^2^ enrolled into the Vitamin D and Lifestyle Intervention for GDM Prevention (DALI) pilot, lifestyle and vitamin D studies of this pan-European multicentre trial, attended for an OGTT during pregnancy. Demographic, anthropometric and metabolic data were collected at enrolment and throughout pregnancy. GDM was diagnosed using IADPSG/WHO 2013 criteria. GDM treatment followed local policies.

**Results:**

The number of women recruited per country ranged from 80 to 217, and the dropout rate was 7.1%. Overall, 39% of women developed GDM during pregnancy, with no significant differences in prevalence across countries. The prevalence of GDM was high (24%; 242/1023) in early pregnancy. Despite interventions used in the DALI study, a further 14% (94/672) had developed GDM when tested at mid gestation (24–28 weeks) and 13% (59/476) of the remaining cohort at late gestation (35–37 weeks). Demographics and lifestyle factors were similar at baseline between women with GDM and those who maintained normal glucose tolerance. Previous GDM (16.5% vs 7.9%, *p* = 0.002), congenital malformations (6.4% vs 3.3%, *p* = 0.045) and a baby with macrosomia (31.4% vs 17.9%, *p* = 0.001) were reported more frequently in those who developed GDM. Significant anthropometric and metabolic differences were already present in early pregnancy between women who developed GDM and those who did not.

**Conclusions/interpretation:**

The prevalence of GDM diagnosed by the IADPSG/WHO 2013 GDM criteria in European pregnant women with a BMI ≥29.0 kg/m^2^ is substantial, and poses a significant health burden to these pregnancies and to the future health of the mother and her offspring. Uniform criteria for GDM diagnosis, supported by robust evidence for the benefits of treatment, are urgently needed to guide modern GDM screening and treatment strategies.

## Introduction

Gestational diabetes mellitus (GDM) is defined as carbohydrate intolerance resulting in hyperglycaemia of variable severity with onset or first recognition during pregnancy, excluding those with diabetes in pregnancy likely to represent overt diabetes mellitus [1]. Women with GDM are more likely to suffer pregnancy complications, and the diagnosis is associated with both immediate and long-term adverse consequences for their offspring [2, 3]. Furthermore, studies investigating postnatal maternal glucose function have shown the prevalence of type 2 diabetes to be as high as 38% in the first year postpartum following GDM, and as high as 60% in women followed for up to 16 years postpartum [4–6].

The reported prevalence of GDM in Europe varies considerably, and in certain populations is reported to occur in more than 20% of pregnancies [7, 8]. Unfortunately, accurate prevalence estimates in Europe are lacking due to highly inconsistent screening and diagnostic criteria both in high-risk women and the general pregnant population [9]. This makes pan-European surveys of GDM very difficult and limits the effects of large-scale GDM prevention and treatment strategies. In 2010, the International Association of Diabetes and Pregnancy Study Groups (IADPSG) developed a consensus statement for a new strategy to diagnose GDM [3]. The chosen cut-off point for glucose on a 75 g OGTT conveyed an OR for adverse outcomes of ≥1.75 compared with women with mean glucose levels at 24–28 weeks in the Hyperglycemia and Neonatal Outcomes study [3, 10]. In 2013, both the WHO and the Endocrine Society revised their guidelines and now advise that the IADPSG criteria should be used for the diagnosis of GDM [1, 11]. The International Federation of Gynecology and Obstetrics also supports this approach [12]. Recently, the IADPSG has indicated that the criteria are not for use in early pregnancy [13], but have not provided criteria for up to 24 weeks’ gestation.

The aim of this study was to use information collected during the Vitamin D and Lifestyle Intervention for Gestational Diabetes Mellitus Prevention (DALI) trial to determine risk factors for GDM development and examine the prevalence of GDM in pregnancy among high-risk women with a prepregnancy BMI ≥29 kg/m^2^ across 11 centres in Europe using the IADPSG/WHO 2013 diagnostic thresholds.

## Methods

The DALI trial was a prospective, multicentre RCT that compared different approaches for preventing GDM progression in overweight/obese (BMI ≥29 kg/m^2^) pregnant women recruited prior to 20 weeks’ gestation [7, 14, 15]. A pilot study was followed by a larger study containing two arms: the lifestyle study and the vitamin D study. While the results of the pilot and lifestyle study are available [14, 15], we await publication of the vitamin D study results. The presented study population consists of women from the pilot, lifestyle and vitamin D cohorts [14, 15]. The DALI study enrolled women from 11 centres in nine European countries, representing north, south, east and west areas of Europe (UK, Ireland, Austria, the Netherlands, Belgium, Denmark [two sites], Italy [two sites], Spain, Poland). Each local research ethics committee approved the study and written informed consent was obtained from all participants. The study was performed in accordance with the principles of the Declaration of Helsinki and is registered in the ISRCTN registry (ISRCTN70595832).

### Participant involvement

Representatives of the target group were interviewed in the developmental stage of the DALI trial about their preferences regarding intervention content, modality, frequency and location. These representatives were not involved in the actual conduct of the study, but participants provided feedback on the burden of the intervention and their experiences with the study in general, as part of a process evaluation. Patient organisations have been actively involved in disseminating the results to the lay public.

### Participants

The main inclusion criteria were: age ≥18 years, singleton pregnancy up to and including 19 + 6 weeks of gestation, BMI ≥29 kg/m^2^ before pregnancy (based on RCT feasibility following a review of European obesity prevalence) and ability to give informed consent. Exclusion criteria included pre-existing diabetes, the need for a complex diet, inability to walk ≥100 m safely and the presence of a significant chronic medical condition or psychiatric disease. Consecutive consenting women undertook a 75 g OGTT at <20 weeks’ gestation, with GDM diagnosed from venous samples using locally available laboratory methods according to the IADPSG/WHO 2013 criteria (fasting plasma glucose ≥5.1 mmol/l, 1 h plasma glucose ≥10.0 mmol/l or 2 h plasma glucose ≥8.5 mmol/l) (IADPSG). Women diagnosed with GDM at baseline were excluded from the RCT (lifestyle and/or vitamin D studies). The remaining participants were retested at mid gestation (24–28 weeks) and, if GDM was not diagnosed, were retested again at late gestation (35–37 weeks) with a further 75 g OGTT using IADPSG/WHO 2013 criteria. Recruitment was conducted between January 2012 and February 2014.

### Assessments

Information regarding demographic, anthropometric and metabolic factors was obtained from all women at enrolment through anthropometric and metabolic measurements and by completion of questionnaires [14]. Both neck and waist circumferences were included in the anthropometric assessments, as prior research has demonstrated a relationship between these measurements and glucose intolerance [16, 17]. A 75 g OGTT was performed, with blood samples for glucose and insulin taken at fasting and at 60 and 120 min after glucose ingestion. HOMA-IR was calculated using glucose and insulin values (fasting plasma glucose level [mmol/l] × fasting insulin level [pmol/l]/135) [18].

### Data analyses

Data were entered into a bespoke web-based electronic database. Descriptive data analysis was performed for all variables. Continuous variables are summarised by mean ± SD and categorical variables by counts and percentages. Comparisons between GDM and normal glucose tolerant (NGT) women were performed using *t* tests and/or ANOVA for continuous data and *χ* ^2^ tests for binary data. For non-normally distributed data, non-parametric tests were used to compare ranks between groups. Multivariate logistic regression models were applied to identify risk factors for GDM diagnosis at any time point adjusting for potential confounders. Variables significant in univariate analysis, as well as those previously identified as risk factors in the literature, were entered into the multivariate model. To avoid collinearity, highly correlated factors were entered in separate models (e.g. BMI, weight, neck and waist circumference). The risk factor with the highest OR was retained in these instances. Factors (at baseline) included age, weight, BMI, neck/waist circumference, marital status, education, parity, employment, alcohol consumption, smoking, (family) history of GDM and previous pregnancy risk factors (congenital malformation, macrosomic baby [>4 kg], previous pregnancy loss, polycystic ovary syndrome [PCOS], chronic hypertension). Statistical analysis was performed using SPSS version 21.0 (SPSS, Chicago, IL, USA). A two-sided *p* value <0.05 was considered statistically significant. No imputations were made for missing data.

## Results

### DALI enrolment

Enrolment occurred across 11 sites in nine European countries. A total of 3544 women were screened and 1023 (28.9%) were enrolled (pilot, lifestyle and vitamin D trials), of whom 73 women (7.1%) dropped out or were lost to follow-up across all sites. Enrolment rates (defined as the percentage of eligible women enrolled per site) among countries ranged from 7.8% to 21.2% (average 11%), giving a wide spread across the European populations (Table 1).

**Table 1:** Enrolment of pregnant women in the DALI pilot, lifestyle and vitamin D studies by country

Country	Number enrolled	Percentage of total trial population
Denmark (two sites)	217	21.2
UK	125	12.2
Italy (two sites)	116	11.3
Austria	110	10.8
Belgium	101	9.9
Spain	99	9.7
Poland	91	8.9
Ireland	84	8.2
Netherlands	80	7.8
TOTAL	1023	100

### Prevalence of GDM

Table 2 outlines the GDM prevalence by period of gestation and country. A total of 395/1023 women were diagnosed with GDM at some time during pregnancy: 242 (61.3%) in early pregnancy, 94 (23.8%) at mid gestation and 59 (14.9%) at late gestation. There was a high prevalence of GDM in early pregnancy (<20 weeks’ gestation), with 242 women (24%) from the overall population of 1023 diagnosed at this point. Five of these women met the criteria for overt diabetes in pregnancy. Of the 242 women diagnosed in early pregnancy, 190 (78.5%) met the diagnostic criteria based on fasting glucose alone (≥5.1 mmol/l). Five women of Asian ethnicity were diagnosed in early pregnancy and all five were diagnosed based on fasting glucose, with two of these women exceeding the glucose cut-off at all three time points. Women diagnosed in early pregnancy were not enrolled in the DALI RCT. There was a spread in the prevalence of GDM in early pregnancy, from a low of 10–11% in the UK and Ireland to a high of 43% in Denmark. A total of 672 women were retested at mid gestation (24–26 weeks), and 94 (14%) had developed GDM despite the interventions of the trial. Once again, there was a spread in prevalence from a low of 8% in Ireland to a high of 21% in Italy. Finally, 476 women who were previously categorised as having NGT (without GDM at the OGTT at 24–26 weeks) completed the final OGTT at 35–37 weeks’ gestation, of whom 59 (13%) had developed GDM despite diet and lifestyle interventions. There was a spread in prevalence from a low of 9% in Italy to a high of 16% in Belgium. Overall, 395 (39%) women fulfilled the IADPSG/WHO 2013 criteria for GDM at any point within the trials. The lowest overall prevalence was in the UK (24%) and the highest prevalence was in Denmark (52%).

**Table 2:** GDM prevalence according to gestation period and country

Country	Participants enrolled, *n*	Participants with GDM, *n* (%)			GDM total, *n* (%)
		Early pregnancy	Mid pregnancy	Late pregnancy	
Denmark	217	93/217 (43)	11/106 (10)	8/77 (10)	112/217 (52)
Belgium	101	21/101 (21)	12/75 (16)	10/61 (16)	43/101 (43)
Spain	99	25/99 (25)	10/65 (15)	6/46 (13)	41/99 (41)
Netherlands	80	27/80 (34)	4/44 (9)	1/18 (5)	32/81 (41)
Poland	91	17/91 (19)	12/68 (18)	7/47 (15)	36/91 (40)
Austria	110	22/110 (20)	12/72 (17)	6/45 (13)	40/110 (36)
Italy	116	16/116 (14)	18/85 (21)	5/54 (9)	39/116 (34)
Ireland	84	9/84 (11)	5/63 (8)	7/47 (15)	21/84 (25)
UK	125	12/125 (10)	10/94 (11)	9/74 (12)	31/125 (24)
TOTAL	1023	242/1023 (24)	94/672 (14)	59/476 (13)	395/1023 (39)

### Demographic and lifestyle factors

Table 3 outlines the demographic and lifestyle factors at baseline for all women enrolled in the DALI RCT according to glucose tolerance status (GDM at any time vs NGT). There was no significant difference in ethnicity between those who maintained NGT and those who developed GDM during the trial. More than 50% of participants reported having a high (university-equivalent) level of education, reflective of the university cities in which recruitment occurred. The percentage of women with a low level of education was similar in those with NGT and GDM at 12.0% and 12.8%, respectively. Overall, 5.2% of women reported consuming alcohol during pregnancy. One third of fathers (33.8%) were active smokers, double the rate reported by mothers (16.2%), with no difference in active smoking between women with NGT and GDM. Nulliparous women accounted for 50.3% of the total cohort.

**Table 3:** Demographic/lifestyle factors of participants at enrolment

Variable (%)	All women	NGT	GDM^a^	*p* value^b^
Ethnicity				
European	86.3	86.4	86.2	NS
Non-European	13.7	13.6	13.8	NS
Education				
High	55.2	56.9	52.4	NS
Medium	32.5	31.1	34.3	NS
Low	12.3	12.0	12.8	NS
Living with a partner	93.4	93.8	91.8	NS
Employment status				
Working	77.0	77.1	76.8	NS
Not working	14.3	13.8	15.0	NS
Home duties	8.7	9.1	8.2	NS
Current maternal alcohol consumption	5.2	6.1	3.8	NS
Current maternal smoking	16.2	16.7	15.4	NS
Current paternal smoking	33.8	34.6	32.6	NS
Nulliparous	50.3	48.7	52.9	NS

^a^At any time during pregnancy

^b^Women with NGT vs GDM

### Maternal GDM risk factors

Maternal risk factors for GDM development at enrolment are displayed in Table 4. There were no differences between women with GDM at any time and those with NGT with respect to a history of diabetes in a first-degree relative, PCOS, reported chronic hypertension or a reported previous pregnancy loss. More women with GDM than NGT reported a previous history of GDM (16.5% vs 7.9%, *p* = 0.002), congenital malformations (6.4% vs 3.3%, *p* = 0.045) and a previous macrosomic baby (31.4% vs 17.9%, *p* = 0.001).

**Table 4:** Maternal risk factors for GDM development

Variable (%)	All women	NGT	GDM^a^	*p* value^b^
Diabetes in a first-degree relative	24.6	23.7	27.7	NS
PCOS	10.4	10.3	10.7	NS
Chronic hypertension	13.0	12.8	13.7	NS
Previous GDM^c^	9.8	7.9	16.5	0.002
Previous baby with a congenital malformation^c^	4.0	3.3	6.4	0.045
Previous macrosomic baby^c^	21.0	17.9	31.4	0.001
Previous pregnancy loss^c^	11.1	10.2	14.1	NS

^a^At any time during pregnancy

^b^Women with NGT vs GDM

^c^In women with a previous pregnancy

### Anthropometric/metabolic characteristics prepregnancy and at first assessment

Table 5 shows anthropometric and metabolic measurements among women with NGT and those with early and mid/late pregnancy GDM. Significant differences were observed between women with NGT and those with GDM. Reported mean prepregnancy and enrolment weight and BMI were significantly greater in women with GDM in early pregnancy. Mean waist circumference was significantly different among those with NGT (107.3 cm), those with early GDM (114.4 cm) and those with GDM in mid or late pregnancy (107.1 cm) (*p* = 0.009). Significant differences were also noted among the groups in terms of systolic BP, diastolic BP and heart rate, with higher measurements observed for those women with GDM in early pregnancy. As expected, women with GDM in early pregnancy had higher fasting, 1 h and 2 h glucose levels, but these levels were also significantly higher in women who went on to develop GDM later compared with women with NGT. HOMA-IR at first assessment was significantly higher in those with early vs mid/late GDM, and those with NGT had the lowest level (4.5, 3.6 and 3.0, respectively; *p* < 0.001).

**Table 5:** Anthropometric/metabolic factors prepregnancy and in early pregnancy according to glucose tolerance

Variable	NGT	GDM early pregnancy	GDM mid/late pregnancy	*p* value^a^	*p* value^b^
Age at enrolment (years)	31.9 ± 5.4	32.7 ± 5.1	32.0 ± 5.0	NS	NS
Height at enrolment (cm)	165.7 ± 6.7	165.5 ± 6.1	165.0 ± 7.4	NS	NS
Prepregnancy weight (kg)	92.8 ± 13.5	96.8 ± 16.1	91.0 ± 14.9	<0.001	<0.001
Prepregnancy BMI (kg/m^2^)	33.7 ± 4.2	35.3 ± 5.4	33.4 ± 4.7	<0.001	<0.001
Weight at enrolment (kg)	94.8 ± 13.5	99.3 ± 17.1	93.5 ± 14.7	<0.001	<0.001
BMI at enrolment (kg/m^2^)	34.5 ± 4.2	36.2 ± 5.6	34.2 ± 4.4	<0.001	<0.001
Waist circumference at enrolment (cm)	107.3 ± 10.1	114.4 ± 60.9	107.1 ± 10.1	0.009	NS
Neck circumference at enrolment (cm)	36.3 ± 2.1	37.4 ± 4.9	36.3 ± 2.2	<0.001	0.008
Systolic BP at enrolment (mmHg)	116.4 ± 11.1	118.5 ± 10.1	115.9 ± 10.8	0.03	0.019
Diastolic BP (mmHg)	72.8 ± 8.5	75.1 ± 8.5	73.0 ± 11.8	0.003	0.036^c^
Heart rate at enrolment (beats/min)	79 ± 10.2	82 ± 10.6	81 ± 9.3	0.001	NS
Fasting glucose in early pregnancy (mmol/l)	4.5 ± 0.3	5.2 ± 0.5	4.6 ± 0.3	<0.001	<0.001
1 h glucose in early pregnancy (mmol/l)	6.5 ± 1.4	8.9 ± 2.0	7.5 ± 1.4	<0.001	<0.001
2 h glucose in early pregnancy (mmol/l)	5.7 ± 1.1	7.3 ± 1.7	6.2 ± 1.1	<0.001	<0.001
HOMA-IR	3.0 ± 2.6	4.5 ± 2.7	3.6 ± 3.6	<0.001	0.007

Data are means ± SD

^a^Comparison across the three groups

^b^GDM in early pregnancy vs GDM in mid/late pregnancy

^c^NS when applying Bonferroni adjustment for multiple comparisons

#### Risk of GDM development

Table 6 outlines the results of a multivariate logistic regression analysis to evaluate the OR of GDM development. Independent risk factors for GDM development were nulliparity (OR 1.6, 95% CI 1.0, 2.5), neck circumference at initial visit (OR 1.1, 95% CI 1.0, 1.2), GDM in a previous pregnancy (OR 2.3, 95% CI 1.3, 4.0) and a previous macrosomic baby (OR 1.7, 95% CI 1.1, 2.6).

**Table 6:** Multivariate logistic regression analysis evaluating the risk of GDM (yes/no) at any stage of gestation

Variable	OR	95% CI	*p* value
Nulliparous	1.6	1.0, 2.5	0.032
Neck circumference at initial evaluation (cm)^a^	1.1	1.0, 1.2	0.011
GDM in previous pregnancy	2.3	1.3, 4.0	0.004
Previous macrosomic baby	1.7	1.1, 2.6	0.014

^a^Continuous variable

#### Pregnancy outcomes according to glucose tolerance

Table 7 shows pregnancy outcomes for all women enrolled in the DALI study vs those with a diagnosis of GDM at mid or late pregnancy (those with early GDM were excluded from enrolment) or NGT. No significant differences were found.

**Table 7:** Pregnancy outcomes for women enrolled in the DALI trials according to glucose status

Variable	All women	NGT	GDM mid/late pregnancy^a^	*p* value^b^
Caesarean section	36.2	34.1	40.2	NS
Pre-eclampsia	3.1	2.8	3.8	NS
Pregnancy-induced hypertension	12.2	11.3	14.2	NS
Birthweight ≥4 kg	16.4	15.2	18.5	NS
Birthweight <2.5 kg	3.9	3.4	4.8	NS
Neonatal intensive care unit admission	9.0	9.9	6.6	NS
Gestational age at delivery (weeks)	39.6 ± 5.9	39.5 ± 3.0	39.8 ± 9.6	NS
Birthweight (g)	3468 ± 578	3456 ± 524	3488 ± 657	NS
Birth length (cm)	51.4 ± 3.5	51.4 ± 3.3	51.4 ± 3.8	NS

Data are % or means ± SD

^a^Women with early GDM were excluded from the DALI study, and pregnancy outcomes are therefore unavailable for this group

^b^Women with NGT vs GDM

## Discussion

Using IADPSG/WHO 2013 criteria, we found an overall GDM prevalence of 39%, across early, mid and late gestation among overweight/obese women who were evaluated for inclusion in the DALI trial. Participants were well distributed across 11 European centres, with the largest cohort (21.2%) attending two sites in Denmark. These data from the DALI trial allow for a meaningful interpretation of the European prevalence of GDM, as identical screening was used in 11 centres across nine European countries, using the IADPSG/WHO 2013 criteria. A substantial proportion of women with GDM were identified in early pregnancy (24%), with 14% and 13% of women diagnosed at mid and late pregnancy, respectively. The prevalence of GDM varied across countries, ranging from 24% in the UK to 52% in Denmark. These findings are in contrast to prior prevalence data that included women across all BMI categories and reported an overall prevalence of 2–6%, with a lower prevalence towards the northern Atlantic seaboard of Europe compared with the southern Mediterranean seaboard [8]. This variance is probably due to a lack of screening uniformity in the aforementioned study, lower glucose cut-off points in the IADPSG/WHO 2013 criteria compared with other guidelines and the inclusion of only women with a BMI ≥29 kg/m^2^ in the current study.

Within this group of women at high risk for GDM, there was no variation in demographic or lifestyle factors according to glucose tolerance, and certain previously reported risk factors (prior GDM, prior macrosomia) were associated with GDM in logistic regression analysis. However, other well-established risk factors for GDM, such as a diagnosis of PCOS and diabetes in a first-degree relative, were not significant: this might be due to their mediation by the overwhelming risk effect of obesity and might be different in a population of women with a wider range in prepregnancy BMIs. While there were variations in GDM prevalence among countries, we did not include country as a variable in our analyses, as a categorical variable with nine levels would cost degrees of freedom and our sample size was insufficiently powered for such an analysis. Overall, however, the significant risk of GDM observed among these overweight/obese women is of concern, given the global rise in obesity and the knowledge that both obesity and GDM are independently associated with adverse maternal–fetal outcomes [10, 19]. Furthermore, 70% of obese women with GDM have been reported to develop type 2 diabetes within 15 years of delivery, compared with 30% of lean women with GDM [20, 21]. It is evident that effective policies to reduce obesity levels are necessary, and that this may result in both clinical and economic benefits [22]. Indeed, an initial step may be to ensure universal BMI screening for women of reproductive age. Then, clinicians may be more likely to assist with weight loss and provide appropriate care to identify and reduce secondary complications of increasing BMI [23, 24].

Among women with a BMI ≥29.0 kg/m^2^ in the current study, those with GDM in early pregnancy had a significantly higher weight and BMI than women with NGT and those with GDM in mid/late pregnancy. It is an interesting observation that these differences were limited to women with early GDM, while women with GDM in mid/late pregnancy only were not significantly different from those with NGT in terms of weight and BMI. Similar findings were observed for neck circumference and systolic and diastolic BP. This is useful clinical information and could be used in a ‘risk score’ for early GDM, although we would recommend screening all women with a BMI ≥29.0 kg/m^2^. The use of neck circumference is a novel measure and may overcome inaccuracies associated with measuring waist circumference [25]. After excluding women with early GDM, women who went on to develop GDM in mid/late pregnancy in the current study also had elevated glucose levels at baseline compared with those who continued to have NGT. This trend was also evident when examining measures of insulin resistance. While there are limitations to indices such as HOMA-IR, such as racial differences in mixed populations, they act as a reasonable surrogate for the gold standard euglycaemic–hyperinsulinaemic clamp [16, 26]. In this study, women with GDM in early pregnancy had significantly higher HOMA-IR values than those with GDM in later pregnancy or those with NGT, as has been previously reported [27]. These findings support prior work characterising women with early-onset GDM as having higher levels of insulin resistance and those with GDM in later pregnancy as being more similar to women with NGT [28]. Higher BMIs among women with early-onset GDM are felt to at least partially explain this phenomenon.

Although screening practices vary, one commonly used approach is to screen the majority of women for GDM at 24–28 weeks’ gestation, with earlier testing reserved for those with risk factors [29]. However, almost a quarter of our cohort was positive for GDM in early pregnancy (<20 weeks’ gestation). While the diagnostic cut-off values for GDM in early pregnancy are controversial [9, 13], our findings indicate that there is some evidence that early screening in obese women may be warranted. As the majority of these women were captured using fasting glucose alone, there may be a role for using this measure rather than completing a full OGTT. Screening is of particular importance in the presence of the additional risk factors we found to be independently associated with GDM development at any stage of gestation. On multivariate analysis, a history of GDM in a previous pregnancy was the strongest independent risk factor identified (OR 2.3). This was followed by a history of having a macrosomic baby (OR 1.7). Future work should consider the use of large for gestational age as an alternative risk factor to macrosomia, as the former can be customised to take into account the specific population under evaluation. Additional independent risk factors included nulliparity (OR 1.6) and a larger neck circumference on initial evaluation (OR 1.1).

Women in this study were diagnosed according to the IADPSG/WHO 2013 criteria [1, 3]. While international practices vary, this approach adopted by the DALI study is in keeping with recommendations from the European Board & College of Obstetrics and Gynaecology and the International Federations of Gynecology and Obstetrics [9, 30]. Due to a lower fasting glucose cut-off of 5.1 mmol/l, use of the IADPSG/WHO 2013 criteria results in an increased prevalence of GDM compared with alternative diagnostic criteria; however, the use of these criteria is supported by data revealing that women with fasting plasma glucose levels of 5.1–5.5 mmol/l have an increased risk of adverse outcomes [31]. It is hoped that there will be a move towards uniformity in the diagnosis of GDM across Europe in the future. This will stimulate research in the field of GDM and allow more women to receive a timely diagnosis and appropriate treatment for GDM. It is clear that the criteria for diagnosing GDM in early pregnancy need urgent resolution, and studies to delineate these criteria and quantify any benefits from treatment are urgently required.

Limitations of our findings include the fact that women were recruited and consented to join an RCT, which may have resulted in selection bias. In particular, the high rates of women reporting a previous macrosomic baby suggest that there may have been increased recruitment in women who were perceived (or who perceived themselves) to be at higher risk. The drop out rate of 7.1% compares well with that noted in the UPBEAT study, a similar multicentre trial involving complex lifestyle interventions with the aim of reducing GDM incidence [32]. The primary reasons cited for dropping out in the DALI pilot and lifestyle studies included lack of time and pregnancy loss [14, 15]. We do not address the optimal GDM prevention strategy, as women received a variety of lifestyle modifications and vitamin D therapy during the DALI trial, and these are reviewed in the DALI pilot and lifestyle RCT outcomes papers [14, 15]. If the interventions had an impact on individual women then the prevalence of GDM in mid to late pregnancy calculated in the current study may be an underestimate. On the other hand, as discussed in relation to early pregnancy, use of the IADPSG criteria to diagnose additional individuals with GDM in late pregnancy is not validated and the justification for this requires further study. Finally, once diagnosed with GDM, women were managed according to local practice at each site and this could have influenced birth outcomes.

Nevertheless, the women in this study were from a wide spectrum of European countries and the data were carefully collected, recorded and analysed to give an accurate overview of GDM patterns in this population. This work fills an important gap in the literature and highlights the high prevalence of GDM associated with obesity in a European population.

In conclusion, the overall prevalence of GDM diagnosed by the IADPSG/WHO 2013 GDM criteria in European pregnant women with a BMI ≥29 kg/m^2^ participating in the DALI study was high (39%). We have identified a number of independent risk factors for GDM development in this cohort. It is evident that there is an urgent need for uniform strategies for screening and diagnosing GDM, along with effective preventive interventions.

## Abbreviations

DALI: Vitamin D and Lifestyle Intervention for Gestational Diabetes Mellitus Prevention study
EBCOG: European Board and College of Obstetrics and Gynaecology
GDM: Gestational diabetes mellitus
IADPSG: International Association of the Diabetes and Pregnancy Study Groups
NGT: Normal glucose tolerance
PCOS: Polycystic ovary syndrome
